# An effective 5-fluorouracil, levofolinate, and oxaliplatin therapy for recurrent breast cancer: a case report

**DOI:** 10.1186/1752-1947-8-234

**Published:** 2014-06-26

**Authors:** Makoto Takahashi, Koichiro Niwa, Shun Ishiyama, Kiichi Sugimoto, Hiromitsu Komiyama, Yukihiro Yaginuma, Yutaka Kojima, Michitoshi Goto, Atsushi Okuzawa, Yuichi Tomiki, Kazuhiro Sakamoto

**Affiliations:** 1 Department of Coloproctological Surgery, Juntendo University Faculty of Medicine, 3-1-3 Hongo, Bunkyo-ku, Tokyo 113-8431, Japan

**Keywords:** Breast cancer, Colorectal cancer, FOLFOX

## Abstract

**Introduction:**

Therapy comprising 5-fluorouracil, levofolinate, and oxaliplatin is currently the most common chemotherapy for colorectal cancer. We experienced a successful case of advanced colon cancer and recurrent breast cancer with 5-fluorouracil, levofolinate, and oxaliplatin therapy.

**Case presentation:**

A 43-year-old Japanese woman who had already undergone surgery three times for bilateral breast cancer was admitted to our hospital for the treatment of advanced transverse colon cancer. Preoperative computed tomography demonstrated a swollen lymph node at her right upper clavicle, and fine-needle aspiration biopsy of the lymph node showed that it was a metastasis from the breast cancer. A laparoscopic-assisted colectomy was performed and the pathology demonstrated that the final stage was IIIC (T4aN2aM0, Union for International Cancer Control, 7th edition). The pathological findings and immunohistochemistry showed that the transverse colon tumor was not a metastatic lesion from the breast cancer, but was a *de novo* colon cancer. Chemotherapy was necessary for both the recurrent breast cancer and the Stage IIIC colon cancer. Therapy of 5-fluorouracil, levofolinate, and oxaliplatin was administered; the therapy included 5-fluorouracil, which is considered to be effective for both colon and breast cancer. After two courses of 5-fluorouracil, levofolinate, and oxaliplatin, the lymph node began to shrink and almost completely disappeared after eight courses of 5-fluorouracil, levofolinate, and oxaliplatin.

**Conclusion:**

We surmise that 5-fluorouracil, levofolinate, and oxaliplatin have the potential to provide a good response for tumors that are sensitive to fluorinated pyrimidine and platinum-containing anticancer drugs such as breast cancer.

## Introduction

A combination therapy of three drugs, 5-fluorouracil (5FU), levofolinate (l-LV) and oxaliplatin (L-OHP; FOLFOX) plays an important role in chemotherapy for colon cancer [1]. We have experienced a successful case in which FOLFOX therapy, as postoperative adjuvant chemotherapy for locally advanced colon cancer, was also effective for the upper cervical lymph node metastasis of a recurrent breast cancer.

## Case presentation

A 43-year-old Japanese woman underwent a physical examination as part of a screening test. She had a notable medical history (Table 1). She had undergone treatment for breast cancer three times. Firstly, at the age of 29 years, she underwent quadrantectomy (Bq) and axillary lymphadenectomy (Ax) for left breast cancer. The pathological findings showed Stage IIA (T2N0M0, TNM Classification Union for International Cancer Control, 7th edition [2]). The postoperative therapy included radiation and uracil-tegafur (UFT) + tamoxifen. Secondly, at the age of 39 years, she underwent Bq and Ax for right breast cancer. The pathological findings showed Stage IIA (T1cN1aM0). The postoperative therapy included radiation and cyclophosphamide, epirubicin, and 5FU (CEF), followed by paclitaxel + trastuzumab. Thirdly, at the age of 43 years, she underwent mastectomy and Ax for left breast cancer. The pathological findings showed Stage IIA (T2N0M0).

**Table 1:** Operative history of our case

**No. of surgery**	**Age (years)**	**Location**	**Procedure**	**Stage**	**Immunohistochemical staining of surgical specimen**					**Adjuvant therapy**
					**ER**	**PR**	**HER2**	**CK7**	**CK20**	
1	29	Lt-breast	Bq+Ax	IIA	(+)	(+)	(+)	(+)	(-)	Rad, UFT+TAM
2	39	Rt-breast	Bq+Ax	IIA	(-)	(-)	(+++)	(+)	(±)	Rad, CEF, PTX+Tmab
3	43	Lt-breast	Bt+Ax	IIA	(-)	(-)	(-)	(+)	(-)	
4	43	T/C	LAC	IIIC	(-)	(-)	(-)	(+)	(+)	FOLFOX

Ax: Axillary lymphadenectomy, Bq: Quadrantectomy, Bt: Mastectomy, CEF: Cyclophosphamide + epirubicin + 5-fluorouracil, CK: Cytokeratin, ER: Estrogen receptor, FOLFOX: 5-fluorouracil, levofolinate, and oxaliplatin, HER2: Human epidermal growth factor receptor 2, LAC: Laparoscopic-assisted colectomy, Lt: Left, PR: Progesterone receptor, PTX: Paclitaxel, Rad: Postoperative radiation therapy, Rt: Right, Stage: TNM Classification Union for International Cancer Control 7th edition, TAM: Tamoxifen, T/C: Transverse colon, Tmab: Trastuzumab, UFT: Uracil-tegafur. Operative history showing the operative procedure, Stage, the outcome of immunohistochemical staining, and postoperative therapies.

**Figure 1:**
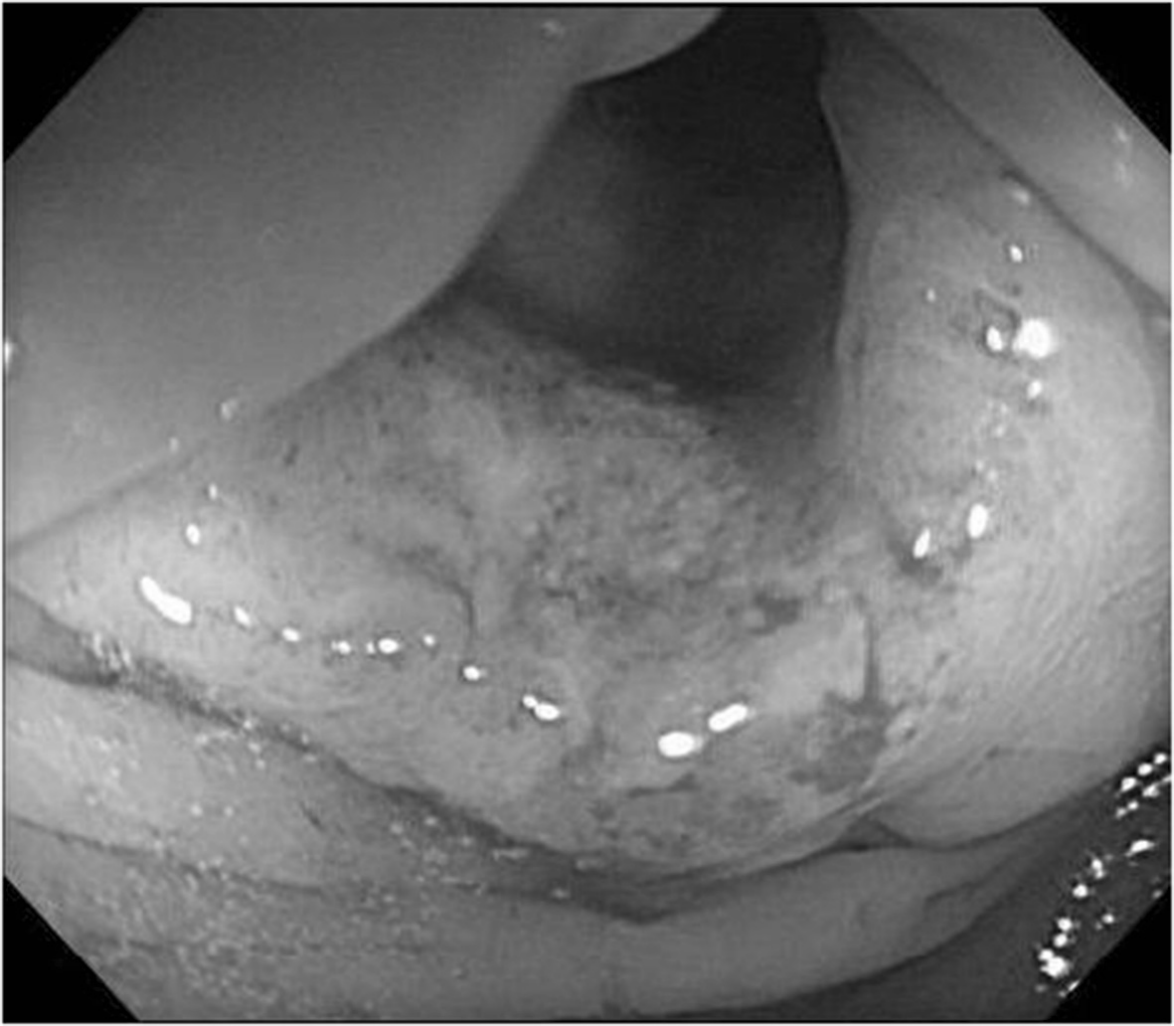
Colonoscopy shows a tumor lesion with severe stenosis at the transverse colon.

**Figure 2:**
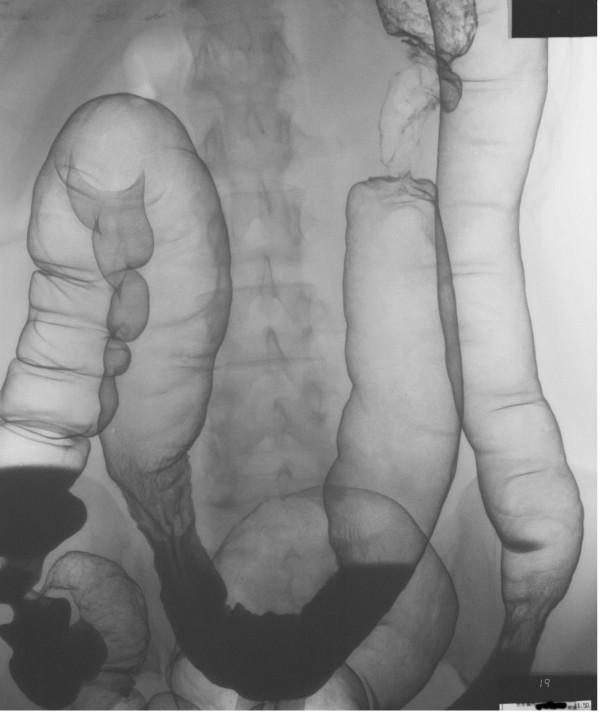
Barium enema shows an apple core sign at the transverse colon.

**Figure 3:**
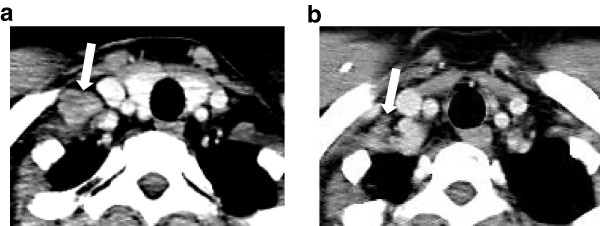
**Cervical computed tomography demonstrates a swollen supraclavicular lymph node.** The size decreased from 20mm (**a**: arrow) to 5mm (**b**: arrow) after FOLFOX.

**Figure 4:**
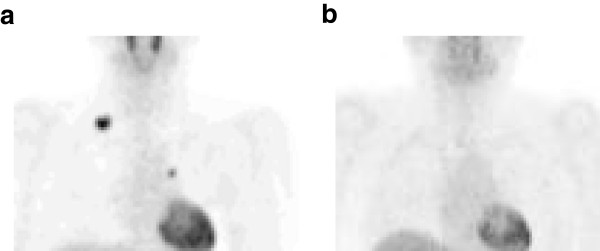
**Positron emission tomography scan. (a)** Positron emission tomography scan shows some ^18^F-fluorodeoxyglucose uptake in lesions at the supraclavicular region and parasternal region. **(b)** However, these lesions vanished completely after 5-fluorouracil, levofolinate, and oxaliplatin.

**Figure 5:**
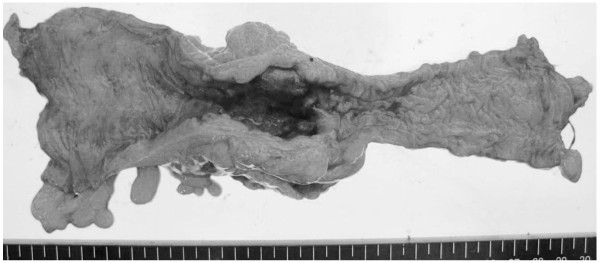
Macroscopic finding of resected specimen shows the diffuse invasive type, 50×42mm in size.

Three months after discharge following the third operation, she visited our department because she was found positive for fecal occult blood during a screening test. Colonoscopy demonstrated a tumor lesion in her transverse colon with severe stenosis that prevented the endoscope from passing through the tumor (Figure 1). The biopsy finding indicated a Group V, poorly differentiated adenocarcinoma. A barium enema examination showed circumferential stenosis (50mm in size) in the transverse colon (Figure 2). Cervical to pelvic computed tomography (CT) revealed a swollen lymph node (size: 21×20mm) in her right supraclavicular region (Figure 3a), abnormal thickness of her transverse colon, and lymphadenopathy around the tumor, and no metastasis in her lungs or liver. ^18^F-fluorodeoxyglucose positron emission tomography (FDG-PET) showed increased FDG uptake in her right supraclavicular region, parasternal region and in her transverse colon (Figure 4a). Neck and parasternal ultrasonography demonstrated lymphadenopathy (22×19×12mm) in her right supraclavicular region and several parasternal lymphadenopathies, approximately 10mm in size. Aspiration biopsy and cytology were performed for her right supraclavicular lymph node and led to the diagnosis of Class V adenocarcinoma, which was very similar to the breast cancer tissues from the third surgery. Therefore, she was also diagnosed as having recurrent breast cancer. There were no abnormalities found in laboratory tests, including tumor markers for colon and breast cancer: carcinoembryonic antigen, cancer antigen (CA) 19–9, p53 antibody, CA 15–3, and human epidermal growth factor receptor 2. Consequently, she was diagnosed as having transverse colon cancer (T3N1H0 cStage IIIA) and recurrent breast cancer, and underwent surgery for resection of the transverse colon cancer. A laparoscopic-assisted partial colectomy was performed. The surgical finding was Stage IIIB (T4aN1M0) R0. The pathological findings showed Stage IIIC (T4aN2aM0), and the histological type was moderately differentiated adenocarcinoma (Tub2), the venous invasion and lymphatic invasion were both positive, and the tumor size was 50×42mm (Figure 5). Immunohistochemical (IHC) staining was performed on the three breast cancer specimens and the colon cancer tissue, considering the possibility of metastatic colon cancer from breast cancer. IHC staining showed that the colon lesion alone was cytokeratin 20 positive (Table 1). Therefore the colon lesion was ultimately diagnosed as a primary colon cancer. IHC staining was difficult in the right supraclavicular lymph node due to the small amount of biopsy tissue available. Although she developed fever after the surgery, she recovered with no significant events, and was discharged on the 23^rd^ postoperative day. As chemotherapy was required to treat both the recurrent breast cancer and the Stage IIIC colon cancer, FOLFOX regimen including 5FU was selected to treat both cancers, because 5FU had been approved for both cancers in Japan. She was started on modified FOLFOX6 (5-FU, 400mg/m^2^ bolus infusion, followed by l-LV 200mg/m^2^ and L-OHP 85mg/m^2^ given concomitantly over 2 hours followed by 5 FU 2, 400mg/m^2^ continuous infusion over 46 hours). She received 12 cycles of FOLFOX every 2 weeks over the course of 6 months with 4 failures of L-OHP. Based on the Common Terminology Criteria for Adverse Events Version 4.0 [3] she experienced grade 1 nausea that did not require any treatment. When she exhibited grade 1 increase of aspartate aminotransferase, grade 2 increase of alanine aminotransferase, and grade 1 increase of γ-glutamyl transpeptidase, the chemotherapy was delayed for 2 weeks. She also exhibited grade 2 peripheral sensory neuropathy due to L-OHP-induced neurotoxicity after administration of the eighth cycle of FOLFOX, and the neuropathy persisted for more than 14 days. L-OHP was subsequently omitted from the 9th to 12th cycles of treatment. She had no grade 3 or 4 adverse events. On occasion she had treatment delays for personal reasons. The right supraclavicular lymph node was not palpable at the end of the second course. Marked reduction of the lymph node was observed with CT and ultrasonography at the end of the eighth course and no abnormal FDG uptake was detected by PET at the end of the twelfth course (Figures 3b and 4b). CT demonstrated the significant reduction of the lymph node size from 20mm to 5mm, and a partial response was then achieved according to new Response Evaluation Criteria in Solid Tumors (version 1.1) [4]. Recurrence of breast cancer has not been observed 1.5 years after the colon cancer surgery and chemotherapy is ongoing.

## Discussion

The medications to treat breast cancer include anticancer drugs, hormone drugs and molecular target drugs, and they are commonly used as monotherapies or in combination [5]. Of the anticancer drugs, anthracyclines and taxanes have played a central role. While fluorinated pyrimidines have been administered to patients with breast cancer, they are not administered mainly as key drugs, but more often as adjuvant chemotherapy in combination chemotherapy, including cyclophosphamide, methotrexate and 5FU (CMF) and CEF. Injectable, oral, ointments and suppositories of fluorinated pyrimidines have been developed. In particular, the current oral anticancer drugs have provided a good response rate against breast cancer (capecitabine, tegafur-gimeracil-oteracil potassium) [6]. In this case, we recommended adjuvant chemotherapy after curative colon cancer resection, because of the pathological diagnosis as Stage IIIC. At the same time, chemotherapy for the recurrent breast cancer was also needed. Thus, we decided to utilize the FOLFOX regimen including 5 FU, a fluorinated pyrimidine anticancer drug, as a protocol that was applicable to both colon and breast cancer. FOLFOX is a regimen that includes a combination of three drugs, 5FU, l-LV and L-OHP, and a large dose of 5FU can be administered by intravenous bolus and continuous intravenous infusion. By administering 5FU instead of uracil, which is incorporated in the nucleic acid synthesis of cancer cells, a ternary complex (TC) is formed with a folate-related substance, l-LV, and thymidylate synthase, thus, inhibiting deoxyribonucleic acid (DNA) synthesis within the cancer cells. As a result, the combination of 5FU and l-LV should increase the antitumor effect in tumors compared with 5FU monotherapy. Folate concentrations are the highest 2 hours after l-LV administration. Therefore, intravenous bolus of 5FU at that time results in the production of high levels of the TC, and 46-hour continuous intravenous infusion of 5FU maintains the TC level for a long time and enhances the inhibition of DNA synthesis to induce anticancer effects [7]. However, the platinum-derivative L-OHP produces crosslinks between DNA purines and inhibits DNA synthesis, resulting in anticancer effects [8]. FOLFOX is a regimen that improves the response rate through a combination of 5FU+l-LV and L-OHP. FOLFOX is currently specific to colon cancer and is used for unresectable or metastatic colon cancer, as well as adjuvant therapy after advanced colon cancer surgery; playing an important role in chemotherapy for colon cancer with a good response rate. However, the platinum-containing anticancer drugs, platinum-derivatives, are not applicable for the treatment of breast cancer in Japan. However, several recent studies have reported that L-OHP has a good response rate in the treatment of breast cancer. For example, some studies of the combination of L-OHP with 5FU and l-LV for recurrent breast cancer have been conducted in Europe and the United States of America and have confirmed the safety and efficacy in a Phase II study [9-11]. While this patient had previously been treated with fluorinated pyrimidine anticancer drugs, such as 5FU and UFT, we expected that the addition of l-LV might increase the anticancer effect. Furthermore, FOLFOX often provides a good response in patients who are resistant to fluorinated pyrimidine anticancer drugs or 5FU+l-LV for colorectal cancer and gastric cancer [12,13]. Based on these considerations, we chose FOLFOX and consequently achieved a good response.

## Conclusions

We treated a patient with Stage IIIC colon cancer that relapsed after surgery for breast cancer, using FOLFOX as a postoperative adjuvant chemotherapy and demonstrated a successful response for recurrent breast cancer. Thus, we surmise that FOLFOX has the potential to provide a good response for tumors that are sensitive to fluorinated pyrimidine and platinum-containing anticancer drugs.

## Consent

Written informed consent was obtained from the patient for publication of this case report and accompanying images. A copy of the written consent is available for review by the Editor-in-Chief of this journal.

## Competing interests

The authors declare that they have no competing interests.

## Authors’ contributions

MT was a major contributor in writing the manuscript. YT and KSa performed the 1st breast surgery. MT, KN, MG, and KSa performed the colon surgery. MT and KSa prepared the manuscript and performed the literature search. YT, AO, MG and YK revised the manuscript. YY, HK, KSu, SI and KN treated the patient. All authors read and approved the final manuscript.
